# The impact of the genetic background in a patient with papillary thyroid cancer and familial adenomatous polyposis

**DOI:** 10.20945/2359-3997000000439

**Published:** 2022-03-08

**Authors:** Guilherme Augusto Barcelos Domingues, Marina Malta Letro Kizys, Carolina Castro Porto Silva Janovsky, Rui Monteiro de Barros Maciel, Magnus Régios Dias-da-Silva, João Roberto Maciel Martins, Cleber Pinto Camacho, Lucas Leite Cunha

**Affiliations:** 1 Universidade Federal de São Paulo Divisão de Endocrinologia Laboratório de Endocrinologia Molecular e Translacional São Paulo SP Brasil Laboratório de Endocrinologia Molecular e Translacional, Divisão de Endocrinologia, Universidade Federal de São Paulo, São Paulo, SP, Brasil; 2 Universidade Nove de Julho Divisão de Pós-Graduação Médica Laboratório de Inovação Molecular e Biotecnologia São Paulo SP Brasil Laboratório de Inovação Molecular e Biotecnologia, Divisão de Pós-Graduação Médica, Universidade Nove de Julho, São Paulo, SP, Brasil

## Abstract

Thyroid cancer is the most common endocrine malignancy, and papillary thyroid carcinoma (PTC) is the main subtype. The cribriform morular variant is a histological phenotype of PTC characterized by its relationship with familial adenomatous polyposis (FAP). Description of the case: We report the genetic assessment of a 20-year-old female patient diagnosed with a cribriform-morular variant of PTC and FAP. We aimed to assess the genetic background of the reported patient, looking for variants that would help us explain the predisposition to tumorigenesis. Genomic DNA was extracted from peripheral blood lymphocytes, and whole exome sequencing was performed. We applied an overrepresentation and gene-set enrichment analysis to look for an accumulation of effects of variants in multiple genes at the genome. We found an overrepresentation of single nucleotide variants (SNVs) in extracellular matrix interactions and cell adhesion genes. Underrepresentation of SNVs in genes related to the regulation of autophagy and cell cycle control was also observed. We hypothesize that the package of alterations of our patient may help to explain why she presented colonic manifestations and thyroid cancer. Our findings suggest that multiple variants with minor impact, when considered together, may be helpful to characterize one particular clinical condition.

## INTRODUCTION

Thyroid cancer is the most common endocrine malignancy, and papillary thyroid carcinoma (PTC) is the main subtype (1).

The cribriform morular variant is a histological phenotype of PTC characterized by its relationship with familial adenomatous polyposis (FAP) (2). FAP is a rare dominant autosomal syndrome found in 1 in 10,000 people in the United States (3). It is possible that patients diagnosed with multiple thyroid cancer nodules would benefit from screening for FAP, particularly in cases where the subtype is the cribriform morular variant (4). Although the relationship between cribriform morular variants is well established, the genetic mechanisms by which FAP predisposes patients to thyroid neoplasia are not well established (5).

Herein, we present a case report in which the diagnosis of a thyroid nodule triggered genetic scrutiny for FAP. We further investigated the overrepresentation and gene-set enrichment analysis to look for an accumulation of the effects of variants in multiple genes at the genome.

## DESCRIPTION OF THE CASE

This study was conducted with the patient's informed written consent and in accordance with the ethics committee of Hospital São Paulo, Federal University of São Paulo (N. 14228713.1.0000.5505). We report the case of a 20-year-old female patient admitted to the thyroid outpatient clinic of our University Hospital (February 2008) due to thyroid nodules. Physical examination evidenced a palpable cervical nodule, and thyroid ultrasonography showed two nodular lesions (1.7 x 1.3 x 1.0 cm sized and 1.5 x 1.1 x 1.0 cm sized). Fine needle aspiration cytology suggested that the dominant nodule was compatible with PTC. Family history investigation revealed that her mother had colorectal carcinoma, and her aunt and cousin had colon cancer.

She underwent total thyroidectomy in March 2008, and pathologic examination confirmed a PTC tumor, 1.5 cm in size, on the right side. Microscopically, the lesion presented multiple growth patterns, including fascicular spindled cells and squamoid islands (morules) scattered throughout the tissue. Immunohistochemistry revealed diffuse positive expression of TTF-1 (SPT24 clone), negative expression of thyroglobulin (DAK-Tg6 clone), and diffuse positive expression of β-catenin (17C2 clone), suggesting the diagnosis of a cribriform-morular variant of PTC. Postsurgery cervical ultrasonography did not show any suspicious lymph nodes, and the thyroid bed was metastasis-free. After surgery, the patient developed hypothyroidism (TSH 42 mIU/L, TG 1.7 ng/mL, both anti-thyroperoxidase and anti-thyroglobulin antibody negatives) and was submitted to actinic ablation with 150 mCi of I131. Posttreatment total body scans failed to show residual or recurrent lesions. She was enrolled in routine follow-up in our institution with periodic neck ultrasound and measurement of serum TSH, thyroglobulin, and anti-thyroglobulin antibodies. She remains relapse-free thus far.

Since the patient had an intriguing family history of colon/colorectal carcinoma, she underwent a proctological examination and rectosigmoidoscopy (in November 2009), which evidenced rectal polyps. On colonoscopy, the rectum and cecum presented multiple sessile polyps (more than one hundred), two to five mm in size, with healthy boundaries. Pathological examination revealed multiple tubular adenomas with low-grade dysplasia. Histological and clinical findings suggested the diagnosis of FAP, and she underwent a total colectomy. We summarized her family for genetic counseling and requested informed consent.

## DISCUSSION

FAP is a hereditary neoplastic syndrome caused by an autosomal mutation of the APC (adenomatosis polyposis coli) gene. Characteristically, multiple noncancerous polyps tend to occur in the colon at an earlier than average age. If untreated, malignant transformation may occur. Patients with FAP present an increased lifetime risk of thyroid cancer and a cribriform-morular variant of PTC (5). However, not all patients with FAP will develop a cribriform-morular variant of PTC. Thus, we further investigated the genetic background of the reported patient looking for variants that would help us to explain the predisposition to the cribriform-morular variant of PTC in patients with FAP.

We extracted genomic DNA from peripheral blood leukocytes of the index case as previously described (6) to perform whole exome sequencing (WES). EdgeBio (Gaithersburg, USA) conducted the WES using the SureSelect v5 Human All Exon Kit (Agilent Technologies, Santa Clara, USA) and Illumina HiSeq 2000 (Aros Applied Biotechnology, Denmark). They aligned the 100 bp paired-end reads to the hg18 human reference genome. Variations were categorized into several functional classes using prediction algorithms, PolyPhen2 (http://genetics.bwh.harvard.edu/pph2/) and SIFT (http://sift.jcvi.org/).

To unveil single nucleotide variants (SNVs) and indels that may explain the phenotype of our patient, EdgeBio called all variants from WES. In total, 115,845 different changes, of which 110,987 were covered ≥10x. We excluded intronic, intergenic, and synonymous variants. According to this annotation, 57,099 coding variants were nonsynonymous, including missense mutations, frameshifts, gain/loss of stop codon, placed on a 3’ or 5’ untranslated region (UTR). We prioritized rare variants not previously described in the dbSNP130, NHLBI Exome Variant Server, and 1000 Genomes databases. We yielded 32,099 SNV or indels variants.

We identified four variants in the APC gene. Interestingly, the patient carries a germline frameshift mutation in APC caused by deleting one adenine at amino acid 418, exon 10. This mutation creates a new stop codon, nine codons downstream, located in the first 15% of the protein. Indeed, SIFT prediction suggests that this indel variant has a damaging effect (SIFT score = 0.858), probably causing nonsense-mediated decay. One variation was a homozygous polymorphism that led to a valine of an aspartic acid (V1822D) change (c.5465T>A (p.Val1822Asp)). This polymorphism is thought to be tolerated according to SIFT (Provean score = 0.59; SIFT score= 0.672) and benign according to PolyPhen. Clinically, V1822D seems to be protective (7). There are two other variations placed at the 3’UTR. No mutation in MYTYH was found, confirming our clinical suspicion of FAP.

One single gene seems insufficient to represent the complexity of the genetic background of our patient. Thus, we looked for an accumulation of effects. First, we performed a clustering analysis of affected genes on pathways. We further investigated the corresponding genes identified in the first step on their enrichment in KEGG pathways (8) with GeneTrail (9). We then performed an overrepresentation analysis (ORA) and gene-set enrichment analysis (GSEA). In ORA, genes of a test set are compared to a reference platform.

On the other hand, on GSEA, a sorted test set can be analyzed without a reference set, testing whether the sorted list of test genes is uniformly distributed considering a functional category. Since many classes are usually tested, the raw p values need to be adjusted for multiple testing. The results were adjusted for the control of the false discovery rate (10).

We analyzed the subcategories of genes mutated according to Pfam domains in proteins coded by respective genes. Table 1 illustrates the distribution of genes found. We observed an overrepresentation of subcategories such as cadherin and cadherin-like domains. Cadherins constitute a superfamily of calcium-dependent cell adhesion molecules that play critical roles in maintaining tissue structure and morphogenesis and are commonly disrupted in various cancers (11).

**Table 1:** Single nucleotide variants distribution according to subcategories of Pfam domains.

Subcategory name	p-value	Expected number of genes	Observed number of genes	Examples of Gene IDs of test set in subcategory
DnaJ domain	<0.001	20.076	2	SEC63, GAK
Cadherin domain	<0.001	48.099	77	*PCDHB8, PCDHB9,FAT2, CELSR1, CDH13,PCDHB10, CDHR2, PCDHB2, DSG1, CLSTN1, CDH12 CDH20*
Fibronectin type III domain	<0.001	60.647	89	*TTN, TNXB, EPHB6, ABI3BP, ROBO2, TNXA, IGFN1, FLRT2, FNDC1OSMR, L1CAM*
von Willebrand factor type D domain	0.001	6.274	15	*MUC4, MUC6, MUC5AC, MUC19, FCGBP, MUC5B, VWDE, ZAN, KCP*
Cadherin-like	0.004	27.605	45	*PCDHB8, PCDHB16, PCDHB9, PCDHB7, PCDHB18, PCDHA9, PCDHB15*
PDZ domain	0.007	58.974	83	*GOPC, DVL1, ERBB2IP, MAGIX, NOS1, SLC9A3R2, STXBP4, PARD6A*
Trypsin Inhibitor like cysteine rich domain	0.011	4.601	11	*MUC6, MUC5AC, MUC19, FCGBP, MUC5B, ZAN, OTOG, VWF, MUC2BMPER*
Immunoglobulin I-set domain	0.014	76.540	102	*HMCN2, TTN, ROBO2, IGSF10, IGFN1, HMCN1, LRIG1, OBSL1, L1CAMHSPG2*
Immunoglobulin C1-set domain	0.032	25.095	39	*HLA-DQB1, HLA-DRB1, HLA-A, HLA-H, IGLV1-44, HLA-C, HLA-BHLA-DRB5, SIRPA, TRGC2*
EGF-like domain	0.034	27.605	42	*NOTCH2NL, FAT2, CRB2, CELSR1, ZAN, NOTCH2, CUBN, HSPG2CELSR2*

Table 2 displays the distribution of genes according to KEGG subcategories. We found an overrepresentation of SNVs in genes related to extracellular matrix interactions, focal adhesion, and cell adhesion molecules. Germline variations in genes related to cellular adhesion may facilitate invasion capacity and represent an increased risk for cancer development (12). In fact, the inheritance of a genome enriched with SNVs in genes related to cellular adhesion may add risk for the development of thyroid cancer (13).

**Table 2:** Single nucleotide variants distribution according to subcategories of KEGG pathway.

Subcategory name	p-value	Expected number of genes	Observed number of genes	Example Gene IDs of test set in subcategory
ECM-receptor interaction	<0.001	34.191	57	*TNXB, LAMA5, COL5A3, LAMA1, HSPG2, ITGA3, ITGA8, COL6A3VTN, LAMA2*
Focal adhesion	<0.001	81.813	113	*SHC4, CTNNB1, VEGFC, CAV2, PAK2, PAK6, PAK7, DIAPH1, SOS2PARVB, SPP1*
Regulation of autophagy	0.011	14.246	4	*ATG4B, ULK2, ULK1, GABARAP*
Antigen processing and presentation	0.015	31.748	47	*HLA-DQB1, HLA-DRB1, KIR2DL4, HLA-A, HLA-C, HLA-B, HLA-DRB5, HLA-DPB1, HLA-DQA1, TAP2*
ABC transporters	0.044	17.909	28	*ABCA7, TAP2, ABCA13, ABCB6, ABCG8, TAP1, ABCC6, ABCG5ABCG1, ABCB5, ABCA5*
Arachidonic acid metabolism	0.044	23.608	35	*CYP2E1, CYP4F2, GGT6, JMJD7-PLA2G4B, PLA2G4B, GPX6, LTC4SALOX12, PTGDS*
Cell adhesion molecules (CAMs)	0.044	54.949	72	*CD226CD6, CD80, CD8B, CDH1, CDH15, CDH2, CLDN14, CLDN8, MAG*
Cell cycle	0.044	52.100	36	*CDK6, SMC1B, CDC27, PRKDC, RBL2, MAD1L1, STAG1, CCND2, ATM, CCNE2, ATR*

**Figure 1:**
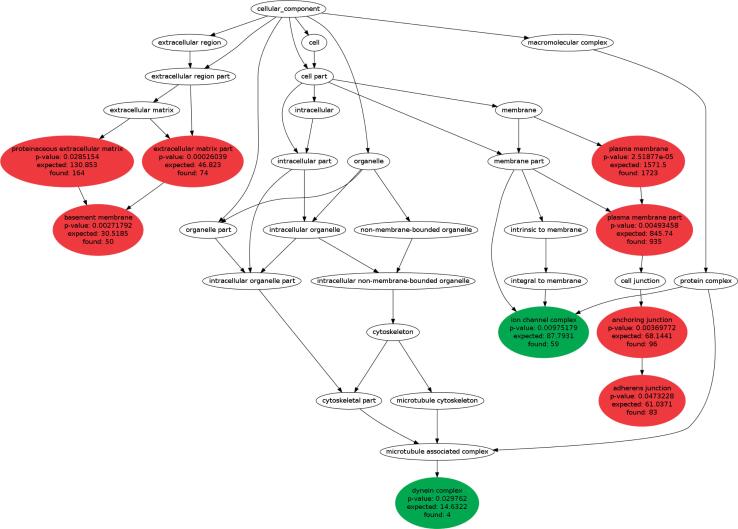
Subcategories of Gene Ontology related to cellular components. Green balloons represent subcategories in which underrepresentation was found. Red balls represent subcategories in which overrepresentation was found. In each balloon, note the expected and observed number of genes.

We also found an underrepresentation of SNVs in genes related to the regulation of autophagy and cell cycle control. Individuals harboring a gene mutation that controls the cell cycle may have an increased risk of developing thyroid cancer (14). Therefore, the package of alterations in our patient may help explain why she presented colonic manifestation and thyroid malignancy. Gene ontology analysis reinforces that our patient harbors a genotypic profile of gene alterations related to tumor promotion and inhibition of tumor restraint, as shown in Figure 1.

**Figure 2:**
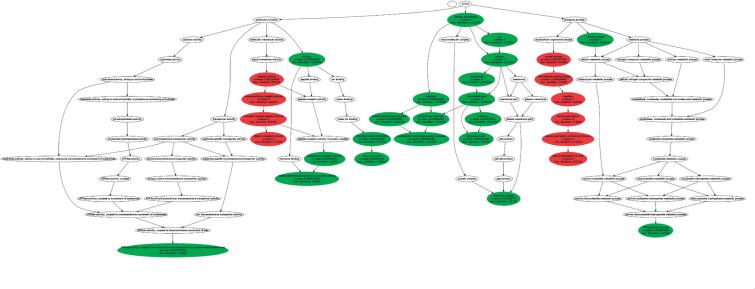
The whole tree of molecular and cellular subcategories of genes mutated in the whole exome of our patient.

To look for enrichment of variations in genes comprising specific biochemical subcategories, we analyzed the genome of our patients using GSEA in GeneTrail. We found olfactory transduction to be one relevant subcategory (p < 0.001). Gene Ontology was able to find 30 significant subcategories, as described in Figure 2. According to Pfam domains, seven subcategories were significantly nonuniformly distributed: seven-transmembrane receptors (p < 0.001), zinc finger (p < 0.001), immunoglobulin C1-set domain (p < 0.001), von Willebrand factor type D domain (p < 0.001), a repeat of unknown function (DUF1220) (p < 0.001), gap junction channel protein cysteine-rich domain (p < 0.001), and Ras family (p < 0.001). Ras family-related proteins are expressed in many different tissues and are involved in tumorigenesis (15).

WES revealed that APC was not the unique mutated gene in the Wnt pathway. We found 572 variants in 216 of those genes. Global minor allele frequency (GMAF) was available for 424 variations. Mean GMAF was 20.9% (range: 0.09-49.95). Mean SIFT prediction was 0.3 (range 0.01-0.98). Half of these variants (50.34%) were classified as nonsynonymous coding, whereas 44.9% were classified as intragenic or coding variants. Only 4.7% of the variants were classified as frameshift mutations or intronic or splice-related variants. We found that only ten genes (ANKRD36C, JUP, ANKRD30BL, ANKRD36, ANKRD20A5P, ANKRD36B, ANKRD20A11P, NPHP3, ANKRD20A7P, CMAHP) were responsible for 31.29% of the variants in the Wnt pathway, suggesting that few genes comprise a hotspot for mutations.

By assessing with scrutiny the WES of our patient, we found variants in genes that mirrored different molecular pathways related to carcinogenesis. These assembled changes helped us understand the phenotypic nuance of the known genetic syndrome, suggesting that multiple variants with minor impact, when considered together, may be helpful to characterize one particular clinical condition. Our study has some limitations. Only one patient was investigated. Thus, we cannot extrapolate our findings to the general population. We did not perform a functional analysis of the genes observed. This may impair major conclusions related to gene functions. However, our study raised some hypotheses that could lead to further genetic investigations. More studies are warranted to test this translational application of WES in a larger cohort of patients with genetic disorders such as FAP.
